# ChroMoS: an integrated web tool for SNP classification, prioritization and functional interpretation

**DOI:** 10.1093/bioinformatics/btt356

**Published:** 2013-06-19

**Authors:** Maxim Barenboim, Thomas Manke

**Affiliations:** Bioinformatics and Deep-Sequencing Unit, Max-Planck Institute of Immunobiology and Epigenetics, 79108 Freiburg, Germany

## Abstract

**Summary:** Genome-wide association studies and re-sequencing projects are revealing an increasing number of disease-associated SNPs, a large fraction of which are non-coding. Although they could have relevance for disease susceptibility and progression, the lack of information about regulatory regions impedes the assessment of their functionality. Here we present a web server, ChroMoS (Chromatin Modified SNPs), which combines genetic and epigenetic data with the goal of facilitating SNPs' classification, prioritization and prediction of their functional consequences. ChroMoS uses a large database of SNPs and chromatin states, but allows a user to provide his/her own genetic information. Based on the SNP classification and interactive prioritization, a user can compute the functional impact of multiple SNPs using two prediction tools, one for differential analysis of transcription factor binding (sTRAP) and another for SNPs with potential impact on binding of miRNAs (MicroSNiPer).

**Availability:** Web server, ChroMoS, is freely available at http://epicenter.immunbio.mpg.de/services/chromos.

**Contact:** barenboim@ie-freiburg.mpg.de or manke@ie-freiburg.mpg.de

## 1 INTRODUCTION

The majority of SNPs from genome-wide association studies (GWAS) and resequencing projects are located in non-coding regions. This impedes the assessment of SNPs' functional effect and disease mechanisms. Handling this plethora of data requires an automated SNP prioritization for initial analysis being followed by more focused functional analysis downstream. In concurrent efforts, epigenetics studies have resulted in many publicly available ChIP-Seq profiles. Ernst *et al.* (2011) designated chromatin states defined by combinatorial patterns of marks using the multivariate hidden Markov model. This allows segmentation of the genome into six annotated general chromatin categories, namely, enhancer, promoter, insulator, transcribed, repressed and inactive states. In turn, these categories are subdivided further into strong, weak and poised states based on their relationship to transcription level of distal and adjacent genes and transcript positional enrichments.

Here we introduce a web server, ChroMoS, which combines genetic and epigenetic data and facilitates SNP classification and prioritization. This integration is achieved through a large database of GWAS results and chromatin annotations, or through direct upload of user-defined data in standardized file formats. Using this data, SNPs can be classified as belonging to various functional categories, such as enhancer or promoter, and prioritized for further analysis. The relationships between SNPs and epigenetic categories were demonstrated by Ernst and colleagues showing that disease-associated SNPs were significantly more likely to be positioned within strong enhancer segments than neutral dbSNP variants. ChroMoS is also integrated with two prediction algorithms, sTRAP and MicroSNiPer, to assess the functional consequences of regulatory SNPs for some transcriptional and post-transcriptional mechanisms, respectively.

## 2 DESCRIPTION OF TOOL

### 2.1 Integrating genetic and epigenetic data

ChroMoS was developed using Perl, PHP and JavaScript. It has a PHP-based web interface and MySQL database as the backend. Perl CGI module runs on Apache 2 web server. ChroMoS is platform-independent and compatible with all major web browsers.

The GWAS data were obtained from National Human Genome Research Institute GWAS catalog accessed on March 15, 2013 (Hindorff *et al., *2009) and were inserted into local MySQL database. BED files with liftover coordinates in hg19 of the chromatin state assignments were downloaded via http://compbio.mit.edu/ENCODE_chromatin_states/ (Ernst *et al.*, 2011). Assigning of SNPs to one of the 15 chromatin states in various cell types is carried out by engaging bedtools intersect (Quinlan and Hall, 2010). Color maps are generated with matrix2png (Pavlidis and Noble, 2003) software. Users can also add their own SNP data through standardized variant call format (VCF).

### 2.2 Data interpretation: sTRAP and MicroSNiPer

A prediction tool, sTRAP (Manke *et al.*, 2010), for differential analysis of transcription factor binding integrated downstream of ChroMoS pipeline allows inputting multiple SNPs (currently 60 SNPs) and runs with the initial threshold of one. After the first run, a user can rerun sTRAP with different thresholds.

A web server, MicroSNiPer (Barenboim *et al.*, 2010), another downstream tool for assessing the differential binding capacity of annotated miRNAs, was updated with dbSNP ver.137 and hg19, and is available at http://epicenter.immunbio.mpg.de/services/microsniper. Large numbers of SNPs can be automatically transferred to MicroSNiPer in VCF and filtered for SNPs positioned in 3′UTRs of RefSeq transcripts (Pruitt *et al.*, 2007). At this step, custom SNPs can be added and filtered before starting a routine MicroSNiPer workflow.

### 2.3 Workflow and usage

**Fig. 1.**
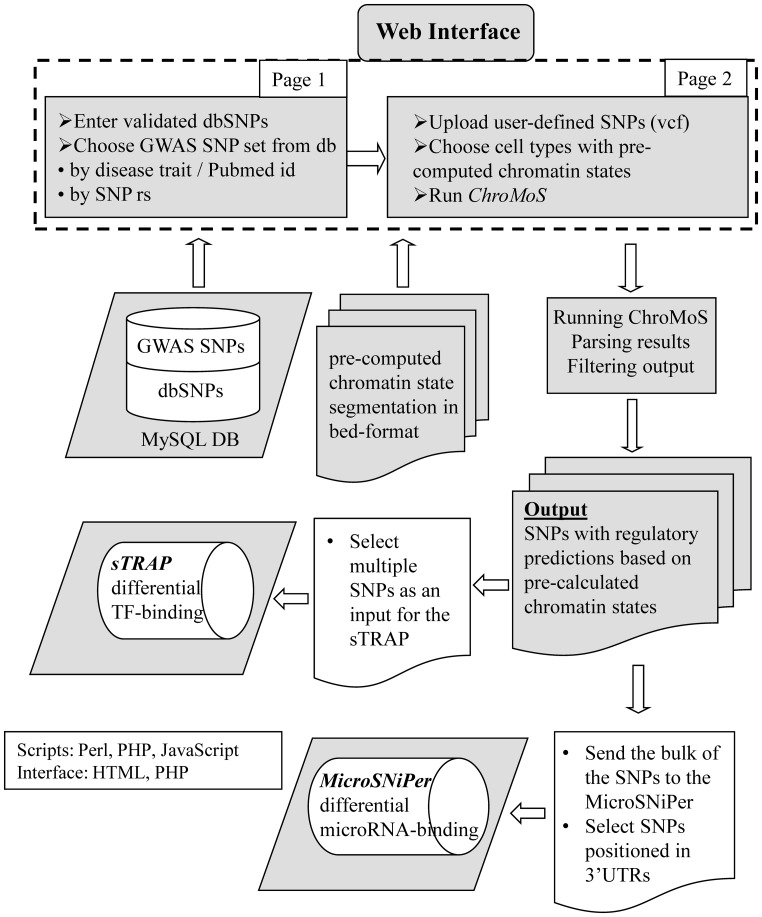
ChroMoS workflow: data integration and prediction tools

A schema of ChroMoS components is presented in Figure 1. At the entry page, a user can enter a list of any validated dbSNP entries or select SNP sets from a large database of GWAS, either by PubMed ID or disease trait, e.g. Crohn’s disease. A user can provide individual rsID number, which will be linked to its respective PubMed IDs. For example, selecting GWA study of Franke *et al.* (2010) by its PMID: 21102463 retrieves 71 SNPs associated with Chron’s disease from local MySQL database. On the second page, a user can add her/his specific SNP dataset in VCF.

Next, the user chooses cell types for which chromatin state information is available and stored in ChroMoS. Currently this includes the 15 states defined by Ernst *et al.* (2011). The output page provides a visualization of the intersection between genetic and epigenetic data and allows the SNP list to be refined according to the chromatin state classes (e.g. enhancer, promoter states). The results can also be downloaded in flat format for sorting and downstream analysis.

Finally, the user-defined SNPs can be selected manually or with customizable filters. For the above example (PMID: 21102463), rs713875 is assigned to a strong enhancer state, pointing to its possible relevance in regulatory mechanism.

After SNP selection, the user can directly run the sTRAP algorithm (Manke *et al.*, 2010) to predict the impact of the SNP on transcription factor binding. Transcription factors with reduced affinity receive a negative log-ratio of *P*-values, and those with increased binding get a positive log-ratio.

If it is suspected that the SNP might be involved in post-transcriptional regulation, the results can be directly transferred to the MicroSNiPer web server (Barenboim *et al.*, 2010), which predicts whether a SNP within a 3′UTR target site will disrupt/eliminate or enhance/create a microRNA binding site.

## 3 UTILITY OF TOOL

To the best of our knowledge, currently there is only one web server, HaploReg (Ward and Kellis, 2012), for exploring annotations of the non-coding genome in conjunction with the GWAS or dbSNP variants. ChroMos has a number of benefits in comparison with this tool. First, it allows for easy and flexible selection of SNPs based on a simple representation of chromatin state categories. Second, the user is not limited to predefined rsID SNPs, but can also evaluate new SNPs not yet contained in the database. Finally, we provide concurrently a direct prediction for the functional outcomes of multiple SNPs.
